# Primary Leiomyosarcoma of the Glans

**DOI:** 10.3389/fonc.2022.851003

**Published:** 2022-05-09

**Authors:** Raimundo Nonato Gois da Costa Junior, Antonio Augusto Lima Teixeira Júnior, Thalita Moura Silva Rocha, Thaís Bastos Moraes Sobrinho, Liseana de Oliveira Barbosa, Rafael Campos Silva, Rita da Graça Carvalhal Frazão Corrêa, Antonio Machado Alencar Junior, Francisco Sergio Moura Silva Nascimento, Syomara Pereira da Costa Melo, José Ribamar Rodrigues Calixto, Gyl Eanes Barros Silva

**Affiliations:** ^1^ Laboratory of Immunofluorescence and Electron Microscopy, University Hospital of the Federal University of Maranhão (HUUFMA), São Luís, Brazil; ^2^ Molecular Pathology Study Group (GEPAM), University Hospital of the Federal University of Maranhão (HUFFMA), São Luís, Brazil; ^3^ Departament of Genetics, Ribeirão Preto Medical School, University of São Paulo Ribeirão Preto Medical School (FMRP/USP), Ribeirão Preto, Brazil

**Keywords:** leiomyosarcoma, penile tumors, immunohistochemistry, histopathological, glans

## Abstract

Penile leiomyosarcoma isz an extremely uncommon entity that rarely occurs in the glans. Due to the limited number of cases described in literature, guidelines regarding non-surgical treatment, prognosis, and management remain equivocal. Among the mesenchymal tumors of the penis, leiomyosarcoma has the highest propensity for recurrence. It originates in the smooth muscle cells from two distinct locations: superficial and deep. The deep subtype is the most aggressive and has the highest potential for metastasis. Surgical treatment should be implemented early and must be locally aggressive. Herein, we present a rare case of a 54-year-old patient with deep localized leiomyosarcoma of the glans, albeit with superficial characteristics. A review of the main histopathological, clinical, immunohistochemical, and therapeutic aspects of this unusual entity is presented.

## Introduction

In the penis, squamous cell carcinoma (SCC) is by far the most common neoplasm, accounting for 95% of all neoplasms. Mesenchymal tumors are extremely rare, representing <5% of all types of malignant tumors (1). According to the World Health Organization, leiomyosarcoma is the second most common penile sarcoma after Kaposi’s sarcoma. The patients’ age at leiomyosarcoma diagnosis ranges from 6 (2) to 80 (1) years, and the most prevalent age group is 42–63 years (mean age, 52 years). Pathologically, leiomyosarcomas are classified into superficial and deep lesions (1, 2). The superficial type usually appears in areas of integumentary support, and the deep type originates from the support structures of the corpora cavernosa and corpus spongiosum (3). We herein report a rare case of leiomyosarcoma of the glans in a 54-year-old patient who underwent surgical resection of the lesion. In addition, we review the histopathological, immunohistochemical, clinical, and therapeutic aspects of this unusual entity.

## Case Report

**Figure 1:**
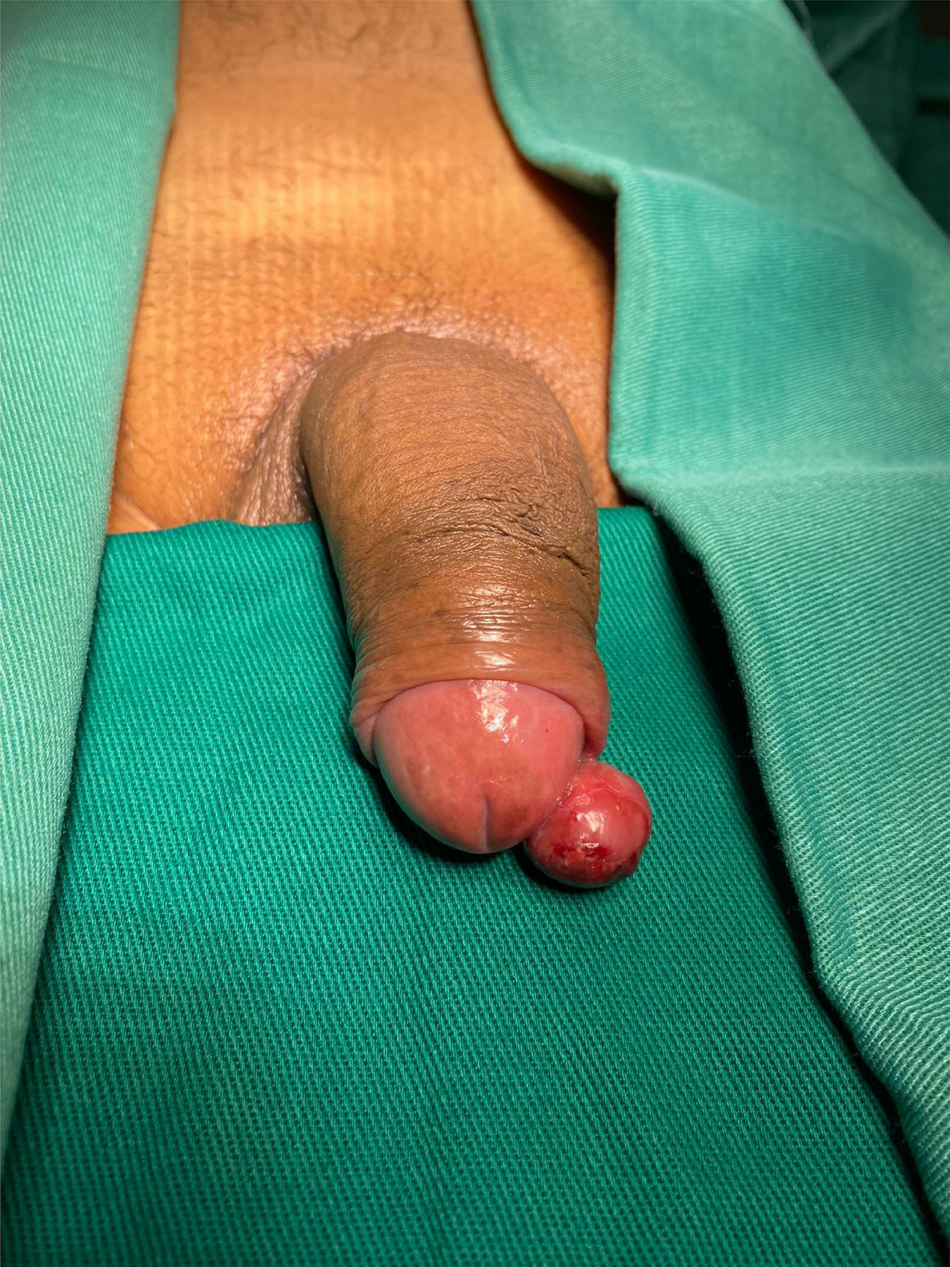
A 54-years-old patient with a pedunculated nodule at the tip of glans that was show to be leiomyosarcoma on histopathological exam.

**Figure 2:**
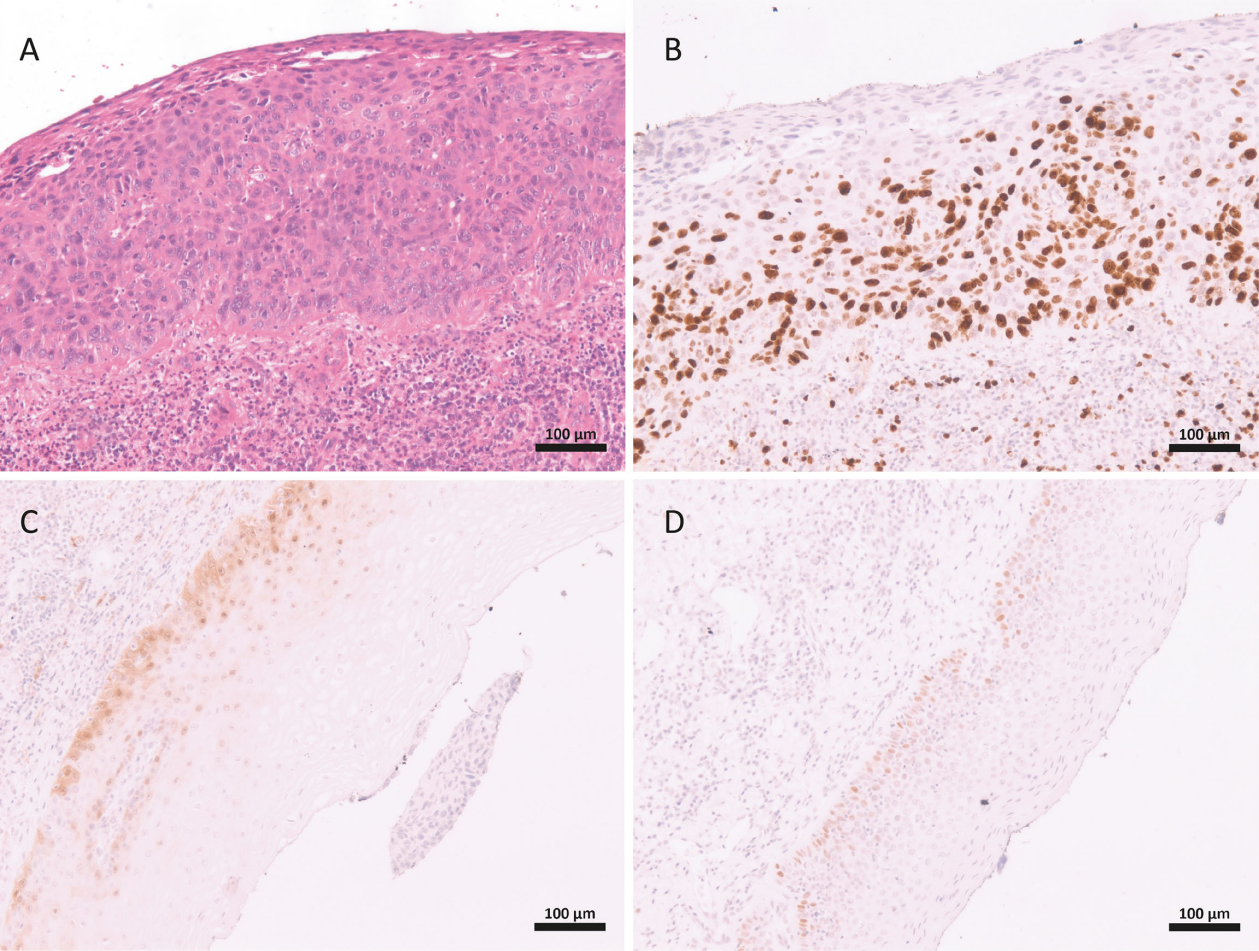
Features of Penile intraepithelial neoplasia in the glans of a patient with leiomyosarcoma. Dysplastic epithelial cell in all squamous layers **(A)**. Immunohistochemistry staing for: Ki67 in suprabasal layers **(B)**, p16 **(C)** and p53 **(D)**.

A 54-year-old male patient visited the urology department with a 6-month history of a “nodule (similar to callus)” on the glans. The lesion was exophytic and grew gradually and painlessly. The patient had no history of bleeding or weight loss. A physical examination identified a nodule at the tip of the penis, measuring 2.0 × 1.0 cm (**Figure 1**). Inguinal lymphadenopathy was not detected. On palpation, the nodule had a soft consistency. The lesion was excised, and histopathological examination of the specimen under a microscope revealed a morphology similar to that of malignant spindle cell neoplasm, with high mitoses and atypical mitoses. The surgical margin had neoplasm. However, neither angiolymphatic nor perineural invasion were observed. The corpus spongiosum, tunica albuginea, corpora cavernosa, and urethra were all free from neoplasia. A focal area compatible with Penile intraepithelial neoplasia (PeIN) was also observed, with epithelial layers showing immunostaining for P16, P53, and Ki67 (**Figure 2**).

**Figure 3:**
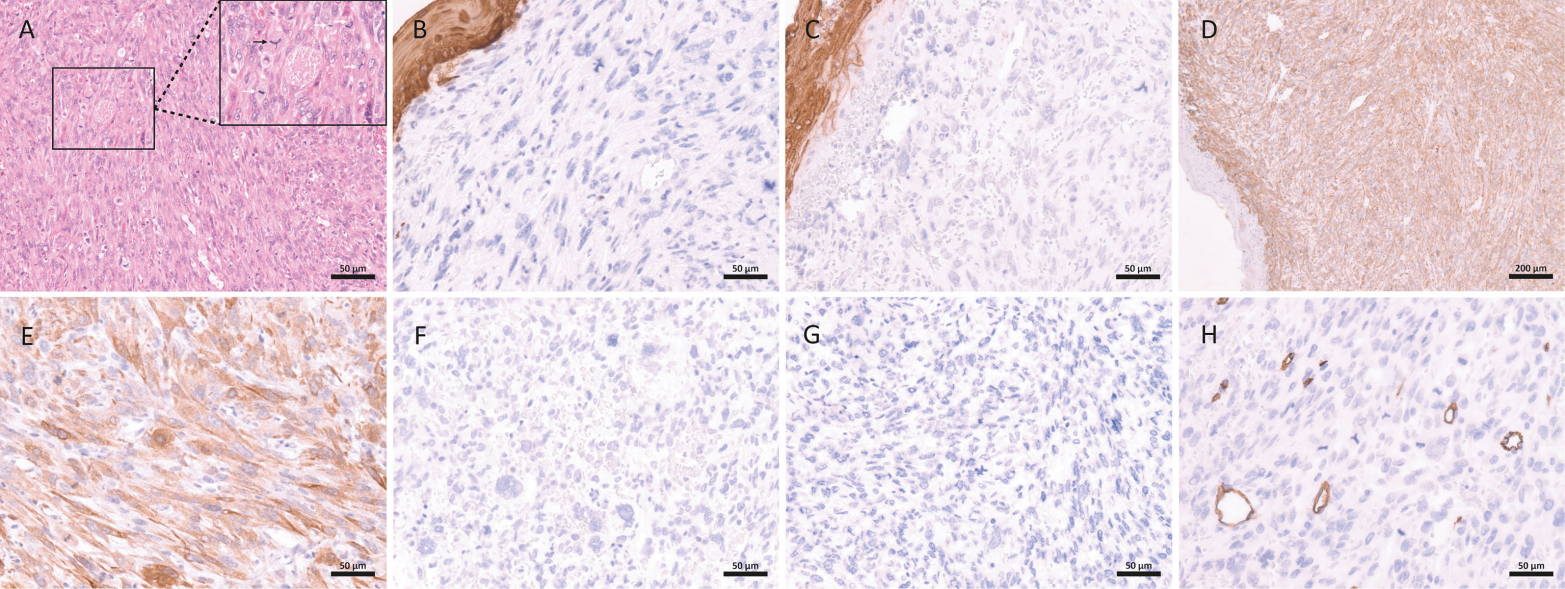
Histopathological and immunohistochemical aspects of leiomyosarcoma of the penis: spindle cell malignant neoplasm with a high number of mitoses (white arrow) and atypical mitoses (black arrow) **(A)**; the neoplasm was negative for epithelial markers: epithelial membrane antigen **(B)** and pan-cytokeratin **(C)**; the immunohistochemical expression was strong and diffuse for smooth muscle markers: alpha-smooth muscle actin **(D)** and calponin **(E)**; the neoplasm is also negative expression for: p16 **(F)**, S100 **(G)** and CD34 **(H)**.

Immunohistochemical tests were positive and diffuse for muscle antigens, specifically smooth muscle actin (SMA), calponin, and HHF35. Tumor cells were negative for, CD34, CD31, v-ets erythroblastosis virus E26 oncogene like isoform 2 (ERG), and FLI1 (**Figure 3**). Polymerase chain reaction assay for human papillomavirus (HPV) in two fresh samples of the invasive tumor showed no viral infection. Eventually, a case of high-grade penile leiomyosarcoma was diagnosed with a focus on PeIN.

At 5 months since presentation, the patient shows no signs of recurrence. The patient has been advised a reoperation to enlarge the compromised surgical margin. However, so far, the patient refuses to resurgery.

## Discussion

Primary mesenchymal tumors of the penis are among the least common tumors of the male genitourinary tract (1, 4, 5). The first case of penile leiomyosarcoma was described by Levi in 1930 (1). The total number of superficial leiomyosarcoma reported cases was 30 in 2013 (6), and although current data regarding the exact number of cases superficial and deep are disparate, there are certainly fewer than 60 cases reported to date (7–9).

There are two types of leiomyosarcomas of the penis that are classified into two clinically and pathologically distinct entities: superficial and deep leiomyosarcomas (1). Superficial leiomyosarcomas usually manifest as lesions in the distal and dorsal regions of the penis. They typically occur in young patients, are asymptomatic, and show slow tumor growth and low metastatic potential but are locally aggressive. The origin of these superficial neoplasms is presumably the piloerector muscle of the skin or smooth muscle elements of the subcutaneous cellular tissue (1, 5).

Deep leiomyosarcomas are most frequently found in the glans and proximal region of the corpora cavernosa and corpus spongiosum and occur in older adult patients. Unlike superficial tumors, these deep lesions have a higher metastatic potential, with a worse prognosis, increased aggressiveness, and a tendency to infiltrate the urethra and produce symptoms, such as urethrocutaneous fistula and urinary obstruction. These deep lesions can result from the progression of initially superficial lesions or from the smooth muscle cells of the corpora cavernosa and corpus spongiosum (5).

Leiomyosarcoma is described as a mesenchymal tumor prone to recurrence, which becomes increasingly histologically undifferentiated after each recurrence (3, 10). Our patient presented with a leiomyosarcoma that behaved like a superficial leiomyosarcoma, but in a region of the penis more characteristic of a deep leiomyosarcoma. This suggests that deep leiomyosarcomas can result from the progression of an initially superficial lesion (5). Additionally, the lesion was located in the glans. Of a total of 30 cases leimyosarcoma superficial reviewed, in only 3 cases, it occurred exclusively in the glans (6). As in our case, the three cases of glans tumors did not show recurrence or metastasis. Despite being considered a deep lesion, leiomyosarcomas that affect the glans seem to behave as superficial lesions.

Differential diagnoses of this neoplasm include sarcomatoid SCC, neurogenic sarcoma, angiosarcoma, fibrosarcoma, and, most importantly, Kaposi’s sarcoma. Immunohistochemistry is essential for a definitive diagnosis. Leiomyosarcoma is distinguished from sarcomatoid carcinoma by its negative immunoreactivity to cytokeratin’s. Kaposi’s sarcoma has a prominent lymphoplasmacytic infiltrate and is immunoreactive for CD31, CD34, and HHV8. Fibrosarcomas are rare in the penis and are smooth muscle actin-negative. In our case, the tumor cells were positive for SMA (strong and diffuse), calponin (strong and diffuse), and HHF35, which are muscle antigens, and negative for pan-cytokeratin (AE1AE3), CK18 and EMA (all epithelial markers), s100 (neural/melanocytic marker), and CD31, CD34, ERG, and FLI1 (all endothelial/vascular markers).

Surprisingly, our patient also had a focal area of PeIN, with immunoreactivity for p16, p53, and Ki67, but with no human papillomavirus (HPV) infection. However, HPV research was not performed around the *in situ* lesions. Due to the high frequency of HPV and associated lesions in our region, the PeIN is likely a fortuitous finding. However, the presence this lesion makes it essential to completely exclude the possibility of the sarcomatoid variant of the SCC through a wide panel of epithelial markers, as performed in our patient. Interestingly, to date, this is the only case of a penile leiomyosarcoma associated with PeIN reported in the literature. Of all cases included in our records from 2004 to date (>600), this is the first case of a non-epithelial neoplasm (11, 12).

Local recurrence is frequent in penile leiomyosarcoma ranging from 23% to 29% of cases depending on the deepness of the primary lesion (3) and if there is no distant metastasis, surgery, if possible, would be the preferential salvage approach. The extension of the procedure (amputation vs partial penectomy) depends on the location and extension of the lesion on the penis an invasion of adjacent tissues and structures. Radiotherapy would be reserved to palliation in inoperable tumors, since leiomyosarcomas are not listed at as the most sensitives sarcomas to radiation therapy (13).

Because of the high risk of distant metastasis (50%) of deep primary tumors (3), in these cases we could consider neoadjuvant chemotherapy as an option in large and more aggressive recurrent lesions in order to select *in vivo* good responders and avoid futile aggressive surgery in patients who would inevitably progress to distant metastatic disease. However, these are data extrapolated from extremities high grade leiomyosarcomas, since there is no available data from prospective studies of chemotherapy in penile leiomyosarcomas dur to its rarity (14, 15).

In conclusion, penile leiomyosarcoma has rarely been described in the literature. Hence, reporting this neoplasm will improve recognition and management of this occurrence. This leiomyosarcoma can behave mildly aggressive, with local growth, and indolent, like a superficial leiomyosarcoma, or more aggressive, with rapid progression, and potentially metastatic, like a deep leiomyosarcomas. An accurate histopathological diagnosis, from macroscopy to microscopy, complemented by immunohistochemistry, is essential to avoid misdiagnosis and, consequently, incorrect treatment, especially in regions with a high incidence of penile cancer, such as in our case. Surgical treatment must be locally aggressive to prevent recurrence.

## Data Availability Statement

The original contributions presented in the study are included in the article/supplementary materials. Further inquiries can be directed to the corresponding author.

## Ethics Statement

The study was reviewed and approved by the Research Ethics Committee on Humans from the University Hospital of the Federal University of Maranhão (CEP-HUUFMA), approval term No. 4.228.789 (CAAE No. 30760420.3.0000.5086). Written informed consent was obtained from the patient for the publication of any potentially identifiable images or data included in this article.

## Author Contributions

RNGdCJ, AALTJ, and GEBS are the principal investigators and wrote the first version of the manuscript. TMSR, TBMS, AMAJ, FSMSN, JRRC, and RCS participated in data collection, care and management of the patient. LdOL and SPdCM performed pathological analysis and interpretation. RdGCFC contribute to critical revision of important intellectual content of the manuscript. All authors read and approved the final manuscript.

## Funding

This study was financed in part by the Coordenação de Aperfeiçoamento de Pessoal de Nível Superior - Brasil (CAPES) - Finance Code 001, Empresa Brasileira de Serviços Hospitalares (EBSERH), and for Fundação de Amparo à Pesquisa e ao Desenvolvimento Científico e Tecnológico do Maranhão (FAPEMA) – Award Numbers 01339/17 and 01184/19.

## Conflict of Interest

The authors declare that the research was conducted in the absence of any commercial or financial relationships that could be construed as a potential conflict of interest.

## Publisher’s Note

All claims expressed in this article are solely those of the authors and do not necessarily represent those of their affiliated organizations, or those of the publisher, the editors and the reviewers. Any product that may be evaluated in this article, or claim that may be made by its manufacturer, is not guaranteed or endorsed by the publisher.
